# Perspective: Covid-19; emerging strategies and material technologies

**DOI:** 10.1007/s42247-021-00173-x

**Published:** 2021-03-17

**Authors:** Jubair Ahmed, Hussain Alenezi, Ursula Edirisinghe, Mohan Edirisinghe

**Affiliations:** 1 Department of Mechanical Engineering, University College London, London, WC1E 7JE UK; 2 Emergency Department, Charing Cross Hospital, Imperial College Healthcare NHS Trust, Fulham Palace Rd, Hammersmith, London, W6 8RF UK

## Abstract

It will be remembered in history as the event that brought the world together with science and technology; the COVID-19 pandemic has allowed for decades worth of progression in both healthcare policies and technology development. It has been a show of unprecedented global health policies ranging from the legal requirement for public facemask use to the use of tough movement restrictions that has bought the world’s economy to its knees. Here, we observe the impact of national lockdowns, facemask usage, and their effect on infection rates. It is clear that healthcare policies alone cannot tackle a pandemic. There is a huge pressure to develop personal protective equipment that not only has the capacity to prevent transmission but also has the ergonomics to be worn for long durations. In this work, we reveal our views and thoughts on the healthcare policies and developing materials and technology strategies that have contributed to reduce the damage of the pandemic, coming from the perspectives of materials scientists and a UK National Health Service consultant doctor.

## Preamble

The Covid-19 pandemic has been a test of resilience for science and technology; it has put pressure and asserted direction on the development of science and technology with rewarding advancements being made at a rapid pace. Healthcare policies have been at the forefront of reducing the collateral damage of the outbreak; these new policies have been unprecedented and their approaches novel. We see the impact of national lockdowns on the infection rates over crucial timeframes; we see the policing of face coverings with an abundance of research into material selection and novel manufacturing. With the pace of the pandemic being so rapid, we see a similar race to build effective protection and preventative measures in the form of advanced face coverings, ventilation aids and indeed vaccines. Here we elucidate our own perspectives on emerging strategies. In particular, key materials and manufacturing strategies in stabilising a large-scale Covid-19 pandemic are outlined and discussed.

## Public strategies

When it comes to measuring the effectiveness of healthcare policies, it becomes almost impossible to de-couple and examine individual policies when numerous are deployed in a short period of time. However, it becomes apparent that some decision-making has a larger effect on the population than others. For example, a recent study has analysed the effect of healthcare policies on the number of Covid-19 cases in the most affected countries in the 3 months leading the start of the global pandemic; it found that lockdown measures were highly effective at suppressing a rise in Covid-19 cases [2]. The findings show that generally, the earlier the lockdown measures were enforced; a less steep of rise in cases was seen. We know that the economic implications of forcing a country into lockdown are vast [11, 13]. At the beginning of the pandemic especially, little is known about the extent of how well the pathogen can spread person-to-person; this means that countries had to make difficult decisions based on the trust of scientific and mathematical models [12, 21]. Data suggests that the countries that did adapt to strict lockdown measures did manage to deviate away from a steep and uncontrollable pandemic propagation trajectory in the beginning.

**Fig. 1:**
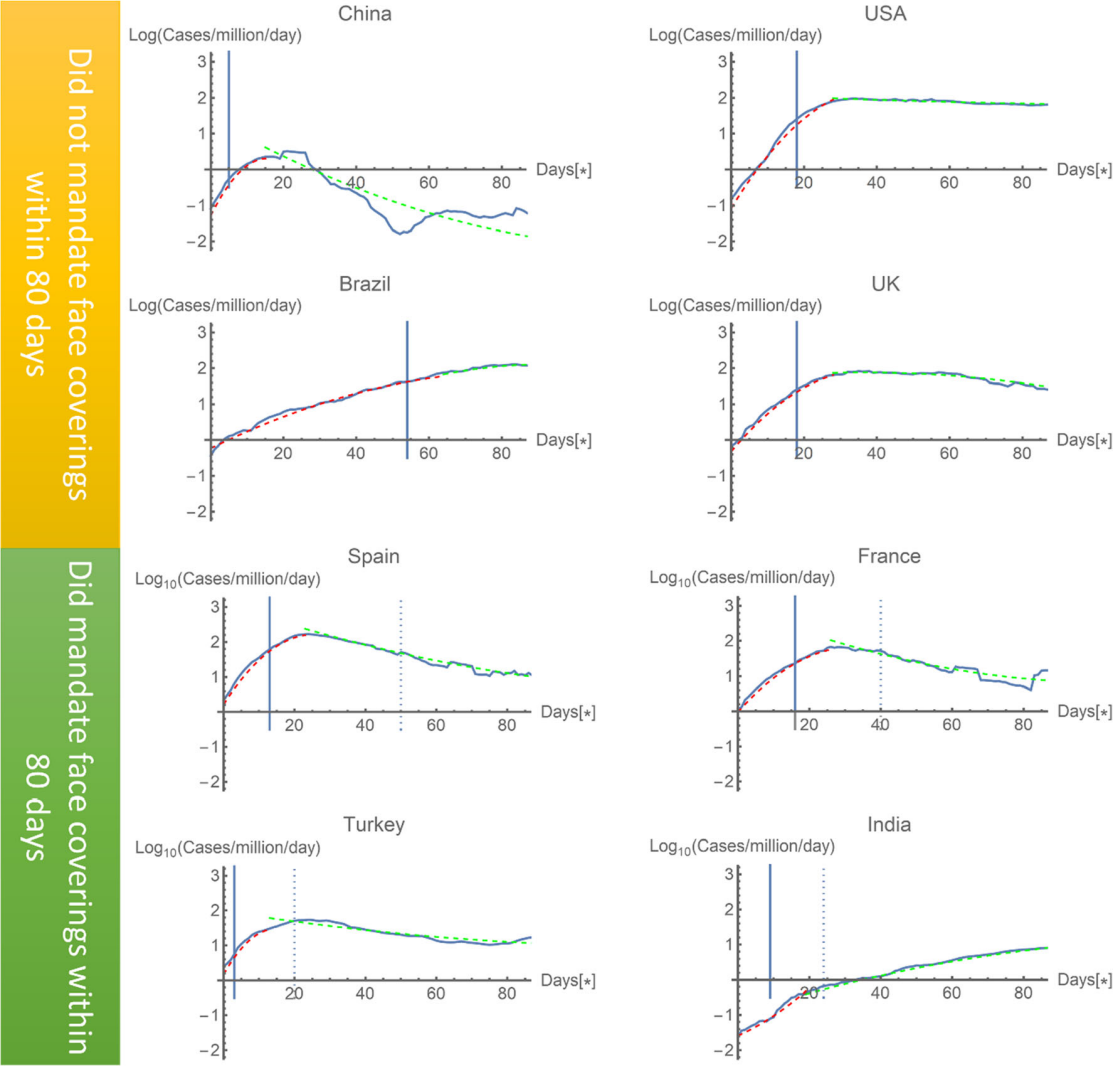
Data collected online from European Centre for Disease Prevention and Control daily database, comparing four countries (China, USA, UK, and Brazil) with another four countries (Spain, France, Turkey, and India) which had different policies on face masks within the initial 3 months of their relative outbreaks. Data began from the 100th reported case in each country. The solid line shows that the reported data and the dashed lines are three-parameter fit smooth curves fitted from day 1 up to day 10 after the imposition of movement restrictions and green dashed curves are a three-parameter fit to the remainder of the data. The relative dates of facemasks being mandate are depicted by a vertical dashed line, where the solid vertical line shows the relative date of the national lockdowns

One of the more controversial public strategies was the mandating of face coverings in public places. In theory, a face covering should be able to protect from an airborne virus that is believed to be predominantly transferred by cough and aerosol droplets [18]. Nevertheless, data on the effectiveness of facemask policies is unclear. Figure 1 shows the data of highly affected countries during the first months of the pandemic; it compares four countries which did and did not mandate a facemask policy within 80 days of the 100th reported case. The solid blue line indicates that when the lockdown began, infections fall off from this activity after the typical incubation period of the virus (8–12 days). The data shows that lockdowns are highly effective in controlling such a viral outbreak; this is due to the less frequent opportunity for the virus to be transmitted. If we then focus on transmission, the data markedly suggests that facemasks cannot reduce infection rates on their own. There are a number of reasons why facemasks can be less effective: the first is due to policies as mentioned in this section and the other is to do with materials and manufacturing technology which will be discussed in the next section. Polices that threaten the freedom of individuals, such as mandating face coverings, can often be ignored by the public. If the chain of wearing face coverings is broken, infection rates can be affected.

Facemasks also have a long-lasting effect on the environment [10]. Made of polymeric materials which have long residence times, their subsequent disposal and degradation will have an impact on the already polluted environment. Hence, materials and manufacturing need to be carefully selected. At least processes using more water-soluble and biodegradable polymers need to be used to mass-produce effective face masks whilst keeping their costs to a minimum.

These findings indicate that mask wearing, combined with other scenarios such as social distancing, regular handwashing with soap, and, in more severe infection rates, lockdown, play a key part in driving infections down [7, 9]. Hence, the speedy tailoring of material morphology and manufacturing to suit is crucial in decelerating the spread of infectious diseases.

## Materials and technologies

Owing to numerous serious shortcomings in the production of face respirators and protective clothes, most operations had halted at the onset of the Covid-19 pandemic. There was shortage in stock for facemasks in global markets [27]. Subsequently, there have been 1.6 million deaths worldwide and Covid-19 has resulted in over 72 million cases [15]; note well that these numbers are, unfortunately, on a steep ascension as a function of immediate time. The scarcity of masks inspired numerous ambitious investigators to develop and manufacture low-cost and efficient respirators with limited resources. Mandating facemasks is not thought to be a highly effective strategy; this is partly due to non-regulation of mask material, meaning ineffective cloth masks can be thought to offer protection, when it is likely they do not [8]. Advanced facemasks such as N95 respirators and other novel designs with improved filtering materials are expected to be much more effective, but high costs will price out the average consumer. Creative prototypes of masks were put forward from various parts of the globe during this Covid-19 pandemic, concentrating on single and multi-use respirators using different materials [6]. For example, multi-layer cloth masks prevent virus transmission to respiratory patients from infected individuals and improve the filtration efficiency; furthermore, methods of ultraviolet and heat application for sterilising respirators have been investigated by other researchers [22].

Respirator wearers had breathing issues because of the decreased amounts of oxygen inhalation resulting from extended use; different systems provide cumbersome exhalation valves [19]. For our contribution, an efficient novel transparent face respirator has been designed and manufactured using product development techniques to achieve the most suitable model using computer-aided design (CAD) tools with the aid of computational fluid dynamics (CFD) to evaluate the mask numerically [3]. In this way, a 75% reduction in filter material was achieved utilising innovative air filtration pathways compared to traditional designs by using the novel square-waveform to improve filter airflow and reduce virus particle inhalation risk. The respirator parts were manufactured using silicone moulding. The materials used for the transparent mainframe and filter cap were clear epoxy resin enabling others to read the lips of users. Silicone was used as the sealant; the respirator can also be produced to custom fit in various sizes where no electrical source is required in manufacturing. This especially benefits developing countries and also as filter material can be substituted manually, reducing costs and waste.

**Fig. 2:**
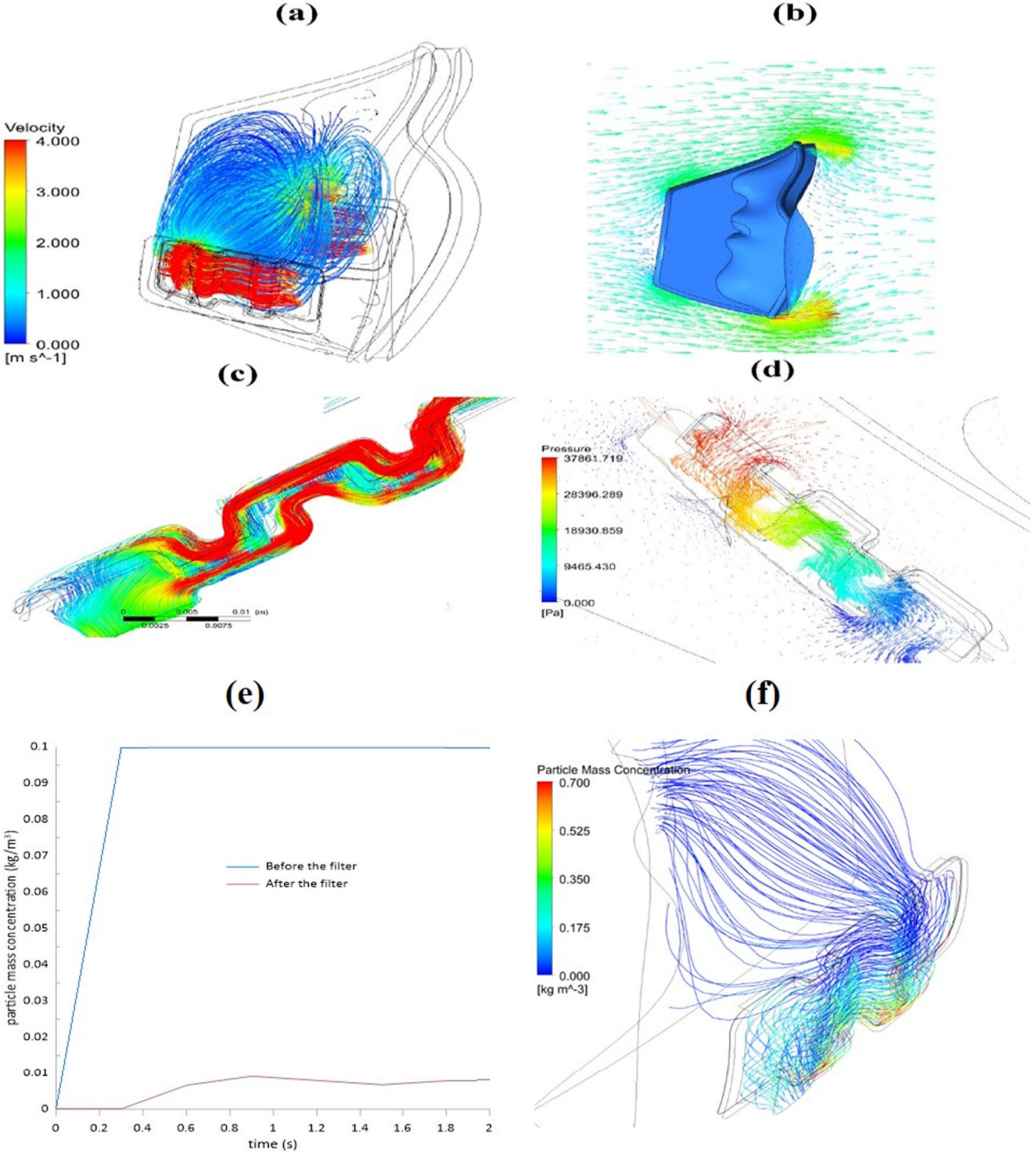
Speed streamlines at one second during natural breathing in the mask’s inner and filter domains, where (**a**), (**b**), and (**c**) are multiple respirator views in which (**d**) indicates the streamlining of pressures within the respirator; (**e**) shows particle mass concentration before and after using the filter over 2s; in (**f**) the streamlines of particle mass concentration across the filter within the mask is shown

Figure 2a displays the speed distribution flow pattern throughout the filter and the internal mask area at one second. Computational fluid dynamics simulation using ANSYS- Fluent 19 was used to understand the pressure, velocity and temperature distribution for resting-level breathing and elevated breathing. The findings indicated that the ventilated air flows within the mask smoothly, leads to higher comfort and is easier for the user to inhale. Owing to the square shape, maximum airspeed is generated in the filter media; therefore, the cross-sectional area decreases, causing turbulent flow. As a consequence, virus particles are pushed into the filter surfaces by airflow. Figure 2b indicates the variation of velocity vector around the mask. The mixture of air and the virus particles hit the mask’s front, allowing the particles to detach from the air which changes directions to get through it. During exhalation at one second, the speed vectors inside the filter domain indicate that the velocity vortex path has shifted dramatically, as shown in Fig. 2c. As indicated, the mixture of air and virus particles have two passages: the first passage is going into the square-wave and passes in front of the filter and the second passage is penetrating the filter, translating to the other side of the filter. The figure shows that most of the air goes in the first pass and hits the walls, increasing the filtering efficiency. The pressure distribution of the whole filter in 1 second is elucidated in Fig. 2d. The internal mask domain is exposed to extreme pressure, and the filter pressure reduces to almost ambient. The effect is that the viscous airflow resistance in the porous filter material rises when the filter stops virus particles from entering. The swirling flow in the masked domain increases the reduction in pressure and thus avoids virus (particles) from passing through the filter. The filtering efficiency is the crucial factor for mask effectiveness. Figure 2e shows the relation between the virus particles mass concentration versus the time before and after using the mask’s filter. It is demonstrated that the particle mass concentration without using the filter is significantly high. Using the filter with a square-wave shape reduced this to near zero; thus, filtering increased to 94%; it is believed that this design was more optimised than a circle shape which could reduce the total filtration area. Figure 2f displays the streamlines of the particle concentration and shows that the concentration increases on the downside of the mask because of the gravity effect.

We have advocated the manufacturing of hybrid polymer fibres, featuring layers with different properties that can help manufacture smart masks [14]. Here, the sheath of the fibres can contain a potent antiviral substance, e.g. copper nanoparticles, whilst the core of the fibres can be a strong polymer which imparts the strength and toughens the mask. This requires upscaling mass production to suit, and work has already been carried out to achieve this [16, 17]. However, in parallel, existing respirators and masks need upgrading to properly inhibit the entry of viral particles, and as with Covid-19, these can be smaller than 100nm.

Alongside the antiviral properties of the nano-sized particles, nanotechnology can also be integrated into COVID-19 detection. Thus, using biomimetic nanoparticles interacting with the virus to enhance their exposure to detection tools can be capitalised on. Gold nanoparticles can aid the development of new and enhanced detection methods suitable for point of care applications [26]. Therefore, nanotechnology can also make a vast difference to controlling the spread of the disease by blocking cell attachment and controlling the spread of the virus. A recent study [23] proposed nanoparticle decoy targets preventing the virus from attaching to cells to reduce the possibilities of developing infection. Nanotechnological principles have helped to pioneer the creation of improved PPE and nanoscale drug delivery systems to overcome COVID-19 [5, 20]. Furthermore, the utilisation of nanotechnology allows for the manipulation of the material’s surface, allowing for hydrophobic and superhydrophobic designs. Hydrophobic outer layers aid to repel infectious droplets from entering the wearer, whilst the inner hydrophobic layer attempts to protect others from self-droplets. The real “game-changing” challenge lies in the development of vaccines, whilst countering virus mutations which will occur as a function of time. Here again, nanotechnology has a crucial role to play and it already contributing to making safe and rapidly effective vaccines against COVID-19 [24].

## Concluding remarks

There are some other important factors which need to be worked upon to fully overcome Covid-19, at least to significantly reduce its impact. One of these is to have a good understanding in surface interactions and viability of coronaviruses, especially in relation to inanimate surfaces and cleaning protocols [4]. The second is in the innovation of novel methods to detect Covid-19 [1, 25]. In pursuing these key lines of intervention, we can truly help defeat Covid-19 and any subsequent outbreaks, as soon as possible.

The Covid-19 pandemic has been of unprecedented magnitude and impact. It has caused a crisis likely to be vividly remembered by all whom have lived through it. It has posed an array of challenges due to complexity of its nature, the unpredictability of symptom severity in different individuals, ease of spread and the practical aspects of its case numbers in terms of pressure on the NHS capacity and public health strategy interventions.

With nearly 100 million cases worldwide and almost 2 million deaths, there have been arguably unparalleled decisions made by government to impose national lockdowns and cancel the world functioning as we know it. Public health strategy can be enforced but has other significant consequences in terms of social isolation and loneliness of the vulnerable, gargantuan economic ramifications and huge impacts on mental health due to life lost and anxiety of the unknown. As well as this, the pandemic has had a knock-on effect on the NHS with mass cancellation of elective work, impacts on medical training with widespread job redeployment to bolster the workforce, as well as emotionally counterintuitive rules allowing little or no visitation in hospital by relatives. This is of course not forgetting the mentally fatiguing impact of working as a healthcare professional in a pandemic where guidelines and protocols evolve daily and new treatments like remdesivir and dexamethasone are refined in a record time. It is also worth mentioning the huge risk that comes from being a medical professional facing such a transmissible and potentially deadly virus, especially in the context of large volumes of patient numbers and aerosol generation. There have been many deaths of healthcare workers during the pandemic. As mentioned previously, modern approaches to high-sensitivity biosensors (such as graphene-based) are revolutionising diagnostics of new diseases and the discovery of novel materials increase the options in material-based healthcare for curing diseases.

Masks have now become integral to daily life not only in the public settings but particularly in the hospital environment. Widely available masks for healthcare workers often do not fit well, are difficult to breathe in and cause irritation leading to skin problems from prolonged use; this can be problematic if the wounds become infected. Materials therefore need to be developed with novel approaches that focus on long-term comfort and ease of breathing as opposed to polypropylene-based masks used now. Whilst many seek for extra protection, using both a mask and a protective shield, little is known about the effectiveness, although it may seem that polymeric screens lull a false sense of high-level protection in many people, outweighing any additional benefits. Whilst wholly necessary in healthcare environments, during the pandemic, they act as barriers for human communication and also need in the ideal design to be practical, economically viable and kind to the environment as well as of course comfortable and effective for the user.

Covid-19 and its magnitude have demanded attention from every human and set a novel challenge for science and technology to step up to in a few short months. The achievements and developments in this short time span have demonstrated great hopes that we are indeed rising to this challenge. In light of vaccine approvals this could be the global panacea that is so desperately needed to halt the pandemic in its tracks and give us time to augment the other strategies employed in our armoury.

## References

[1] Ahmed J (2020) Electrospinning for the manufacture of biosensor components: A mini-review Medical Devices & Sensors n/a:e10136 10.1002/mds3.10136

[2] Ahmed J, Harker A, Edirisinghe M (2020) COVID-19: Facemasks, healthcare policies and risk factors in the crucial initial months of a global pandemic Medical Devices & Sensors:e10120 10.1002/mds3.10120

[3] H. Alenezi, M.E. Cam, M. Edirisinghe, A novel reusable anti-COVID-19 transparent face respirator with optimized airflow. Bio-Design and Manufacturing (2020). 10.1007/s42242-020-00097-1 10.1007/s42242-020-00097-1 PMC7520078 33014512

[4] Aydogdu MO, Altun E, Chung E, Ren G, Homer-Vanniasinkam S, Chen B, Edirisinghe M (2021). Surface interactions and viability of coronaviruses. J. R. Soc. Interface.

[5] V. Bhavana, P. Thakor, S.B. Singh, N.K. Mehra, COVID-19: Pathophysiology, treatment options, nanotechnology approaches, and research agenda to combating the SARS-CoV2 pandemic. Life Sci. **261**, 118336–118336 (2020). 10.1016/j.lfs.2020.118336 10.1016/j.lfs.2020.118336 PMC7443335 32846164

[6] Chao F-L (2020). Face mask designs following novel Coronavirus. J Public Health Res.

[7] Dbouk T, Drikakis D (2020). On coughing and airborne droplet transmission to humans. Phys. Fluids.

[8] Dbouk T, Drikakis D (2020). On respiratory droplets and face masks. Phys. Fluids.

[9] Dbouk T, Drikakis D (2020). Weather impact on airborne coronavirus survival. Phys. Fluids.

[10] Fadare OO, Okoffo ED (2020). Covid-19 face masks: A potential source of microplastic fibers in the environment. Sci. Total Environ..

[11] Gautam S, Hens L (2020) COVID-19: impact by and on the environment, health and economy Environment, Development and Sustainability 22:4953-4954 doi:10.1007/s10668-020-00818-7 10.1007/s10668-020-00818-7 PMC7324289 32837275

[12] Giordano G, Blanchini F, Bruno R, Colaneri P, Di Filippo A, Di Matteo A, Colaneri M (2020). Modelling the COVID-19 epidemic and implementation of population-wide interventions in Italy. Nat. Med..

[13] Gupta M, Abdelmaksoud A, Jafferany M, Lotti T, Sadoughifar R, Goldust M (2020). COVID-19 and economy. Dermatol. Ther..

[14] B. Hays, *Layered hybrid fibers could be used to build anti-viral masks, researchers say* (United Press International, 2020)

[15] John Hopkins University (2020) COVID-19 Dashboard by the Center for Systems Science and Engineering (CSSE). https://coronavirus.jhu.edu/map.html. Accessed 14 Dec. 2020

[16] Mahalingam S, Huo S, Homer-Vanniasinkam S, Edirisinghe M (2020). Generation of core–sheath polymer nanofibers by pressurised gyration. Polymers.

[17] Mahalingam S, Matharu R, Homer-Vanniasinkam S, Edirisinghe M (2020). Current methodologies and approaches for the formation of core–sheath polymer fibers for biomedical applications. Appl. Phys. Rev..

[18] Mittal R, Ni R, Seo J-H (2020). The flow physics of COVID-19. J. Fluid Mech..

[19] Ong JJ (2020). Headaches associated with personal protective equipment–A cross-sectional study among frontline healthcare workers during COVID-19. Headache: The Journal of Head and Face Pain.

[20] P. Paliwal, S. Sargolzaei, S.K. Bhardwaj, V. Bhardwaj V, C. Dixit, A. Kaushik, Grand challenges in bio-nanotechnology to manage the COVID-19 pandemic. Front. Nanotechnol. **2** (2020). 10.3389/fnano.2020.571284

[21] Poletto C, Scarpino SV, Volz EM (2020). Applications of predictive modelling early in the COVID-19 epidemic. The Lancet Digital Health.

[22] Price A et al. (2020) Is the fit of N95 facial masks effected by disinfection? A study of heat and UV disinfection methods using the OSHA protocol fit test medRxiv:2020.2004.2014.20062810 10.1101/2020.04.14.20062810

[23] Rao L, S. Xia, W. Xu, R. Tian, G. Yu, C. Gu, P. Pan, Q.F. Meng, X. Cai, D. Qu, L. Lu, Y. Xie, S. Jiang, X. Chen, Decoy nanoparticles protect against COVID-19 by concurrently adsorbing viruses and inflammatory cytokines. Proc. Natl. Acad. Sci. **117**, 27141 (2020). 10.1073/pnas.2014352117 10.1073/pnas.2014352117 PMC7959535 33024017

[24] M.D. Shin, S. Shukla, Y.H. Chung, V. Beiss, S.K. Chan, O.A. Ortega-Rivera, D.M. Wirth, A. Chen, M. Sack, J.K. Pokorski, N.F. Steinmetz, COVID-19 vaccine development and a potential nanomaterial path forward. Nat. Nanotechnol. **15**, 646–655 (2020). 10.1038/s41565-020-0737-y 10.1038/s41565-020-0737-y 32669664

[25] Tabish TA, Narayan RJ, Edirisinghe M (2020). Rapid and label-free detection of COVID-19 using coherent anti-Stokes Raman scattering microscopy. MRS Communications.

[26] B. Udugama, P. Kadhiresan, H.N. Kozlowski, A. Malekjahani, M. Osborne, V.Y.C. Li, H. Chen, S. Mubareka, J.B. Gubbay, W.C.W. Chan, Diagnosing COVID-19: the disease and tools for detection. ACS Nano. **14**, 3822–3835 (2020). 10.1021/acsnano.0c02624 10.1021/acsnano.0c02624 32223179

[27] Wu H-l, Huang J, Zhang CJP, He Z, Ming W-K (2020) Facemask shortage and the novel coronavirus disease (COVID-19) outbreak: Reflections on public health measures EClinicalMedicine 21:100329 10.1016/j.eclinm.2020.100329 10.1016/j.eclinm.2020.100329 PMC7129293 32292898

